# Human Epidermal Growth Factor Receptor 2 Expression in Prostatic Carcinomas: A Systematic Review and Meta‐Analysis of a Potential Therapeutic Target

**DOI:** 10.1002/pros.70187

**Published:** 2026-04-22

**Authors:** Jacky T. F. Luk, Alex H. Lin, Esther K. C. Cheung, Renald Meçani, Taulant Muka, Jana Nano, Joanna K. M. Ng, Matthew K. L. Chiu, Bryan C. W. Li, Rong Na, Joshua J. X. Li

**Affiliations:** ^1^ Department of Pathology, School of Clinical Medicine The University of Hong Kong Pokfulam Hong Kong SAR; ^2^ Epistudia Bern Switzerland; ^3^ Department of Endocrinology and Diabetology Medical University of Graz Graz Austria; ^4^ Department of Radiation Oncology, TUM School of Medicine and Health, Klinikum Rechts der Isar Technical University of Munich Munich Germany; ^5^ Department of Clinical Oncology, Queen Mary Hospital The University of Hong Kong Pokfulam Hong Kong SAR; ^6^ Department of Medicine, Queen Mary Hospital The University of Hong Kong Pokfulam Hong Kong SAR; ^7^ Department of Surgery, Queen Mary Hospital The University of Hong Kong Pokfulam Hong Kong SAR

**Keywords:** HER2, immunohistochemistry, meta‐analysis, prostate cancer, targeted therapy

## Abstract

**Introduction:**

The clinical relevance of HER2 expression profile in solid cancers has expanded with the evolving landscape of HER2 targeting agents. This systematic review and meta‐analysis aim to detail the prevalence of HER (over)expression in prostate cancer and identify patient/disease subgroups with enriched HER2 overexpression.

**Methods:**

Literature searches of five databases were performed. HER2 scores with cross‐tabulation of clinicopathological parameters were extracted for pooled prevalence and odds ratio analyses. Study quality, heterogeneity, and publication bias were assessed by the CASP checklist, *I* ^2^ index, and LFK index.

**Results:**

In total, 16 studies were included with 1258 cases. There were 669 (53.18%), 269 (21.38%), 250 (19.87%), and 70 (5.56%) of HER2 Scores 0/1/2/3, respectively. Pooled analysis indicated a prevalence of 4.9% (95% CI 3.0%–7.2%) for HER2 Score 3 and 42.0% (95% CI 29.0%–55.6%) for HER2 non‐negative (Scores 1–3). Subgroup analysis comparing HER2 negative (0, 1) and HER2 overexpression (3) indicated higher Gleason score (OR = 0.061, 95% CI: 0.010–0.359, *p* < 0.001), higher disease stage (OR = 0.063, 95% CI: 0.016–0.252, *p* < 0.001) and biopsies from metastatic site (OR: > 100, 95% C.I.: > 100–> 100, *p* < 0.001) favoring HER overexpression, without significant differences for patient age or prostate‐specific antigen (PSA) level. *I* ^2^ was 91.63% with meta‐regression analysis showing study quality as a source of heterogeneity (*p* = 0.043). LFK index was 1.93 (moderate publication bias risk, *p* = 0.054).

**Conclusions:**

HER2 overexpression is rare in prostatic carcinomas but nearly half show HER2 expression of Score 1 or above. Cases with higher Gleason score and advanced disease stage are more likely to overexpress HER2.

## 1 Introduction

The human epidermal growth factor receptor 2 (HER2) is extensively involved in the tumorigenesis of human cancers with promising HER2 related therapeutic agents successfully developed targeting HER expressing cancers [1, 2]. In the case of prostatic carcinoma, the interest on HER2 expression, testing, and treatment has been small as early trials did not demonstrate efficacy for the HER2 inhibitor trastuzumab [1]. The interest in HER2 testing for prostatic carcinoma has been renewed by the availability of antibody–drug conjugates, specifically trastuzumab deruxtecan (TDXd), for metastatic carcinomas [3]. As existing evidence on the HER2 expression profile of prostatic carcinomas is largely limited to single‐center studies of modest scales without representative or consolidated data, a summarization of results would give valuable data with increased generalizability and clinical utility. Such information would be highly useful in exploring systemic treatment options for patients with prostatic carcinoma.

In this systematic review, the prevalence of HER2 overexpression and lower expression levels will be detailed, and further meta‐analysis will be performed to identify patients and/or disease subgroups with enriched HER2 overexpression, aiming to facilitate HER2 related therapeutic decision and planning in patients with prostatic carcinomas.

## 2 Methods

### 2.1 Study Design

The study was designed and conducted following recent guidelines on how to perform a systematic review and meta‐analysis [4, 5], while Preferred Reporting Items for Systematic Reviews and Meta‐Analyses (PRISMA) flowchart was used.

### 2.2 Literature Search

Literature search of articles up to April 19, 2025 was performed, using the bibliographic databases Embase, Medline, PubMed, Scopus, and Web of Science. Key medical subject headings and search terms related to both the biomarker and outcome were used such as “prostate,” “neoplasm,” “cancer,” “carcinoma,” “adenocarcinoma,” “immunohistochemistry,” “HER2,” and “ErbB‐2.” The full search strategy is provided in detail in the Supporting Material S1. Reference lists of the final included studies in the systematic review were screened for eligible studies. EndNote (version 20.1) was used for reference management.

### 2.3 Study Selection

Titles and abstracts of identified articles were screened by two independent reviewers (J. J. X. L. and J. K. M. N.) against predefined inclusion and exclusion criteria and settled discrepancies with discussion or with the opinion of a third reviewer. Studies were deemed eligible if (i) they were designed as randomized clinical trials, retrospective or prospective cohort, case‐control or cross‐sectional studies, or case‐series; (ii) had sample size > 15; (iii) they assessed the expression of HER2 by immunohistochemistry on prostate cancer of human subjects; (iv) they reported the frequency of cases and provided histology and/or clinical data which were evidence of a diagnosis of prostatic carcinomas. Articles were not eligible for inclusion if (i) they were non‐English, review articles, conference abstracts, letters to the editor, editorials or case reports; (ii) they combined categories/scores/ranges of expression level that precluded data extraction; (iii) utilized nonstandardized reporting system(s) for HER2 expression; (iv) combined nonhuman, or nonlesional, nonmalignant or malignant neoplasms other than carcinomas (i.e., lymphomas, sarcomas, and melanomas) and that the results of the HER2 expression of prostatic carcinomas could not be extracted.

### 2.4 Data Extraction and Quality Assessment

The data extraction sheet included study methodology, population characteristics and setting of the population sample, technical procedures, assessment methods, and results of immunohistochemistry. Data extraction was performed by two independent reviewers (J. T. K. L. and J. J. X. L.), and all discrepancies were settled by discussion until a consensus was reached. The Critical Appraisal Skills Programme (CASP) checklist [6] for descriptive or cross sectional studies was used for quality assessment of the included studies.

### 2.5 Statistical Analysis

Extracted test result data (HER2 Scores 0, 1, 2, or 3; or null, negative, equivocal, and positive for expression) were used to calculate the prevalence of each category of HER2 expression. Results were pooled using the random‐effects model. HER2 scores with cross‐tabulation with clinicopathological parameters, if reported in the manuscript, were extracted for meta‐analysis. Statistical heterogeneity was assessed using Higgins' *I* ^2^ statistics, categorizing *I* ^2^ values as follows: < 25% indicated low heterogeneity, between 25% and 50% indicated moderate heterogeneity, and > 50% indicated high heterogeneity. Publication bias was assessed by examination of the DOI plot and its associated Luis Furuya‐Kanamori (LFK) index [7] with Freeman–Tukey transformation [8]. The Freeman–Tukey transformation was applied to all studies, including studies with zero positive cases.

Prespecified random‐effects meta‐regression was performed to investigate potential sources of heterogeneity (including the year of publication, study location, source and assessment method of HER2 immunohistochemistry, and quality of study). Analyses were performed using SPSS (Statistical Product and Service Solutions, version 20) and Python with the following packages—matplotlib, numpy, scipy, sklearn, statsmodels, pandas [9, 10, 11, 12, 13, 14, 15]. A *p* value of < 0.05 (two‐tailed) was considered significant.

## 3 Results

**Figure 1:**
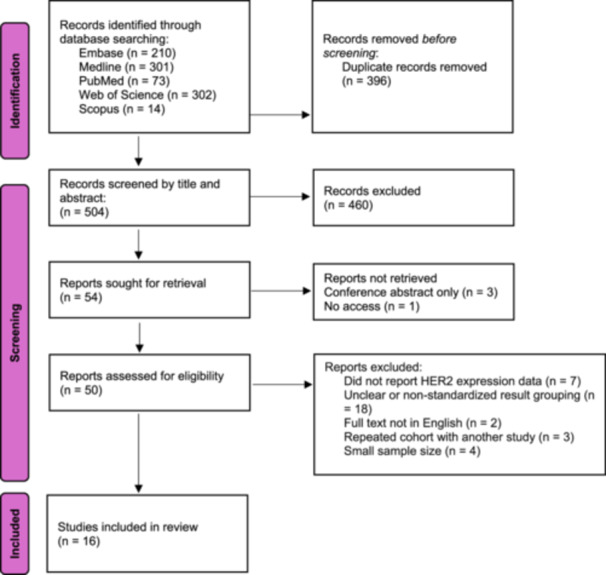
PRISMA flowchart. [Color figure can be viewed at wileyonlinelibrary.com]

Following screening of 504 abstracts, 16 studies (4 case controls, 5 case series, and 7 retrospective cohorts) [16, 17, 18, 19, 20, 21, 22, 23, 24, 25, 26, 27, 28, 29, 30, 31] were included in the final analysis. The selection process is summarized in the PRISMA flowchart (Figure 1). The sample size of the studies ranged from 31 to 231, totaling to 1258 cases. The majority of studies were conducted in the USA (*n* = 6), followed by Turkey (*n* = 3), Italy (*n* = 2), Japan (*n* = 2), China (*n* = 1), Brazil (*n* = 1), and Iran (*n* = 1) (Table 1). In terms of quality assessment, the most common risk of bias among the studies was concerns over measurement accuracy (*n* = 17), followed by subject recruitment (*n* = 7). The design, technical specifications, and detailed CASP assessment of the included studies are listed in Tables S1 and S2.

**Table 1:** HER2 scores of the included studies.

	Year	Country	Total	Score 0	Score 1+	Score 2+	Score 3+
Balta et al. [16]	2024	Turkey	31	12	8	6	5
Estephan et al. [17]	2023	USA	231	95	82	42	12
Dağ and Karabağ [18]	2022	Turkey	59	59	0	0	0
Peixoto et al. [19]	2021	Brazil	146	109	23	14	0
Açikalin et al. [20]	2014	Turkey	59	32	12	9	6
Zahir et al. [21]	2014	Iran	40	16	13	7	4
Tobiume et al. [22]	2011	Japan	102	72	10	14	6
Zhang et al. [23]	2011	China	131	40	8	67	16
Ramieri et al. [24]	2010	Italy	50	46	0	4	0
Montironi and Mazzucchelli [25]	2006	Italy	72	11	33	22	6
Nishio et al. [26]	2006	Japan	49	28	4	11	6
Lara et al. [27]	2002	USA	47	28	14	4	1
Sanchez et al. [28]	2002	USA	38	25	12	1	0
Koeppen et al. [29]	2001	USA	61	46	10	5	0
Osman et al. [30]	2001	USA	103	25	30	42	6
Reese et al. [31]	2001	USA	39	25	10	2	2

**Figure 2:**
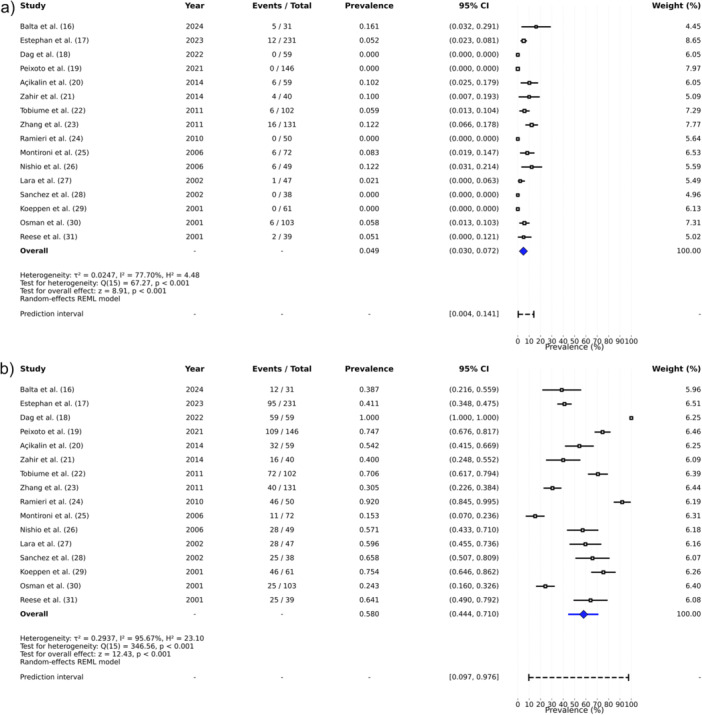
Pooled prevalence of (a) HER2 positive (Score 3) and (b) HER2 null (Score 0) in prostatic carcinoma. [Color figure can be viewed at wileyonlinelibrary.com]

From the 1258 cases, there were 669 (53.18%), 269 (21.38%), 250 (19.87%), and 70 (5.56%) of HER2 Scores 0, 1, 2, and 3, respectively, totaling to 938 (74.56%) negative and equivocal results. Pooled analysis indicated a prevalence of 4.9% (95% CI 3.0%–7.2%) for HER2 Score 3 and 42.0% (95% CI 29.0%–55.6%) for HER2 non‐negative (Scores 1, 2, and 3) (Figure 2).

Subgroup analysis, comparing HER2 negative (0, 1) and HER2 overexpression (3) was performed for Gleason score, prostate‐specific antigen (PSA) level, disease stage, tissue site, and patient age with odds ratio (OR) calculated (Table 2). Gleason score (OR = 0.061, 95% CI: 0.010–0.359, *p* < 0.001), disease stage (OR = 0.063, 95% CI: 0.016–0.252, *p* < 0.001), and tissue site (OR: > 100, 95% CI: > 100–> 100, *p* < 0.001). Higher Gleason score, higher disease stage, and tissue from metastatic sites had higher rates of HER2 overexpression. PSA level and patient age did not show statistically significant correlations with HER2 expression (*p* > 0.05) (Table 2).

**Table 2:** Subgroup analyses.

	HER2 overexpressed	HER2 negative	Odds ratio	*p*
Gleason score				
6	1	113		
≥ 7	25	171	0.061 (0.010, 0.359)	< 0.001
				
PSA level				
< 10 ng/mL	1	7		
≥ 10 ng/mL	15	41	2.561 (0.290, 22.592)	0.667
				
Disease stage				
Local	2	134		
Metastatic^a^	32	135	0.063 (0.016, 0.252)	< 0.001
				
Tissue site				
Prostate	0	89		
Metastatic	12	10	> 100 (> 100, > 100)	< 0.001
				
Patient age				
< 75 years	9	45		
≥ 75 years	7	62	0.565 (0.197, 1.619)	0.297

a Includes regional nodal metastasis.

### 3.1 Heterogeneity and Publication Bias

**Figure 3:**
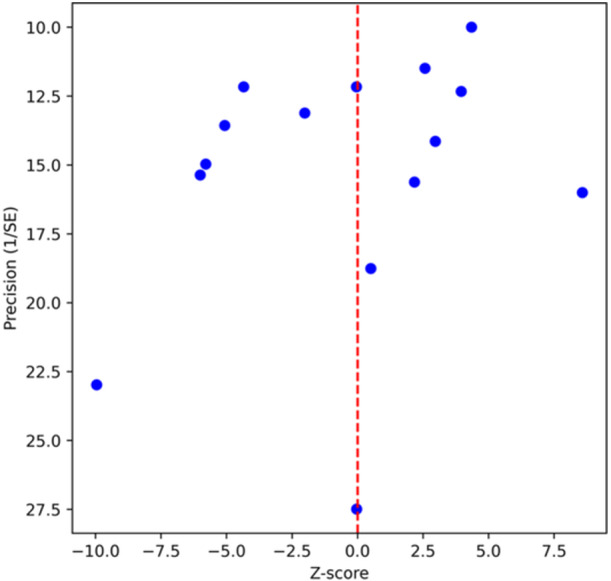
Difference in means of the inverted funnel plot. [Color figure can be viewed at wileyonlinelibrary.com]

*I* ^2^ value was 91.63%, indicating high heterogeneity. Meta‐regression analysis showed study quality (CASP checklist) to be a significant source of heterogeneity (*p* = 0.043), while other factors did not reach statistical significance (*p* > 0.05). Examination of the difference in means of the inverted funnel plot (DOI plot) showed minor asymmetry. The LFK index test with Freeman–Tukey transformation yielded a result of 1.93 (*p* = 0.054), indicating a moderate risk of publication bias (Figure 3).

## 4 Discussion

HER2 is a transmembrane receptor of the epidermal growth factor receptor family with crucial involvement with cell growth, proliferation, and survival [2]. It is of significant clinical interest as HER2 is a highly targetable receptor, with remarkable success in the treatment of breast cancers by HER2 inhibitors, notably trastuzumab [32]. The use of HER2 inhibitors in treatment of prostatic carcinoma is narrow due to its limited efficacy [1], and is overshadowed by the more established treatment modalities in a metastatic context, including androgen deprivation therapy, radiotherapy and chemotherapy [33]. Recent success leading to the approval of TDXd in HER2‐positive solid tumors has renewed interest in HER2 testing and HER2‐related therapies [3, 34]. Its role in prostatic carcinoma is also supported by a report of significant response in HER2‐overexpressed metastatic castration‐resistant case [35]. Furthermore, combination therapy with HER2‐directed antibody–drug conjugates and immune checkpoint inhibitor also appears to be promising direction of development [36, 37] (Table 3).

**Table 3:** Subgroup analyses data.

	Study	HER2 overexpressed	HER2 negative
Gleason score			
6	Estephan et al. [17]	0	75
	Dağ and Karabağ [18]	0	25
	Tobiume et al. [22]	0	1
	Zhang et al. [23]	1	12
≥ 7	Estephan et al. [17]	10	101
	Dağ and Karabağ [18]	0	34
	Zhang et al. [23]	15	36
			
PSA level			
< 10 ng/mL	Zhang et al. [23]	1	7
≥ 10 ng/mL	Zhang et al. [23]	15	41
			
Disease stage			
Local	Estephan et al. [17]	0	72
	Açikalin et al. [20]	0	38
	Zhang et al. [23]	2	24
Metastatic^a^	Estephan et al. [17]	12	105
	Açikalin et al. [20]	6	6
	Zhang et al. [23]	14	24
			
Tissue site			
Prostate	Açikalin et al. [20]	0	38
	Osman et al. [30]	0	51
Metastatic	Açikalin et al. [20]	6	6
	Osman et al. [30]	6	4
			
Patient age			
< 75 years	Dağ and Karabağ [18]	0	34
	Zhang et al. [23]	9	11
≥ 75 years	Dağ and Karabağ [18]	0	25
	Zhang et al. [23]	7	37

a Includes regional nodal metastasis.

The prevalence of HER2 Score 3 was 4.9% among prostatic carcinomas, which is in contrast with the 19% in SEER registry analyses for breast cancer [38]. In addition to the low proportion of cases with HER2 overexpression, the efficacy of Herceptin on prostatic carcinoma was discouraging in clinical trials [1]. However, the indication for TDXd has expanded to not only HER2 Score 3, but also HER2‐low and even ultra‐low cancers [3, 39, 40] with the impressive tumoricidal effects being attributed to “the bystander effects” of TDXd [41]. In this regard, 42.0% of prostatic carcinoma show at least HER2 expression of Score 1, opening greater opportunities for treatment of these patients.

Another point of interest in the landscape of HER2 expression in prostatic carcinomas is identifying groups with enriched expression. As compared to universal HER2 testing for breast cancers, HER2 testing may be better reserved for the second line or for targeted populations. In such cases, identification of patient subgroups enriched in HER2 overexpression prostatic carcinomas would facilitate timely and precision testing. For breast cancers, HER2 expression is associated with younger age, higher histologic grade, and advanced disease stage [42, 43]. Similar associations, including higher Gleason score, higher disease stage, and tissue from metastatic sites, had higher rates of HER2 overexpression from meta‐analysis. Age did not demonstrate correlation with HER2 overexpression.

Limitations of the current study are that all of the included data are retrospective, which is common for biomarker studies that often utilize existing cohorts for further immunostaining [44]. There was heterogeneity and variability among HER2 test methodologies among the included studies that spanned from the year 2001 to 2024, but most adopted the Herceptest (Dako) along with its scoring recommendations, and such was not demonstrated as a significant contributor of heterogeneity in meta‐regression. The definitions of HER2 Score 0 and HER2 null are different [45], and data on this group of patients are not addressed by the current study.

## 5 Conclusions

HER2 overexpression (Score 3) is rare in prostatic carcinomas at 4.9%, but 42.0% of cases show at least HER2 expression of Score 1 or above. Patients with higher Gleason score and advanced disease stage are more likely to have HER2‐overexpressing prostatic carcinomas. Identification of these patients with the expanding indications and repertoire of HER2 related therapies is crucial for population wide and individualized treatment planning.

## Author Contributions

**Jacky T. F. Luk:** data curation, formal analysis, investigation, methodology. **Alex H. Lin:** conceptualization, formal analysis, investigation, methodology, visualization. **Esther K. C. Cheung:** validation, visualization. **Taulant Muka:** methodology, validation, writing – review and editing. **Jana Nano:** validation. **Joanna K. M. Ng:** investigation, validation. **Matthew K. L. Chiu:** validation. **Bryan C. W. Li:** validation. **Rong Na:** validation. **Joshua J. X. Li:** conceptualization, formal analysis, methodology, investigation, writing – review and editing.

## Funding

The authors have nothing to report.

## Conflicts of Interest

The authors declare no conflicts of interest.

## Supporting information

Supporting File.

## Data Availability

The complete data set supporting the findings of this study is available in the manuscript tables and Supporting Materials. The code used is published in GitHub (https://github.com/Joshua-Li-Lab/prostate_her2_meta). Further enquiries can be directed to the corresponding author.
