# Supplementary material for: Human Epidermal Growth Factor Receptor 2 Expression in Prostatic Carcinomas: A Systematic Review and Meta‐Analysis of a Potential Therapeutic Target
Source: Prostate. 2026 Apr 22;86(9):1061–8. doi: 10.1002/pros.70187 (PMC13172941; doi:10.1002/pros.70187)
Supplement: Supplementary file 1 — Supporting File. [file PROS-86-1061-s001.docx]

Supplementary Table 1. Assessment of the included studies

|  | Study type | Antibody clone | Assessment method |
| --- | --- | --- | --- |
| Balta et al. | Case series | Not specified | Other |
| Estephan et al. | Case control | Ventana, 4B5 | Other |
| Dag et al. | Case series | Cell marque, EP3 | Other |
| Peixoto et al. | Retrospective cohort | Dako Herceptest | HercepTest scoring criteria |
| Açikalin et al. | Case series | Dako Herceptest | HercepTest scoring criteria |
| Zahir et al. | Case series | Dako Herceptest | HercepTest scoring criteria |
| Tobiume et al. | Retrospective cohort | Dako Herceptest | HercepTest scoring criteria |
| Zhang et al. | Retrospective cohort | Dako Herceptest | HercepTest scoring criteria |
| Ramieri et al. | Case control | Ventana, 4B5 | ASCO/CAP 2007 |
| Montironi et al. | Case control | Dako Herceptest | HercepTest scoring criteria |
| Nishio et al. | Retrospective cohort | Dako Herceptest | HercepTest scoring criteria |
| Lara et al. | Retrospective cohort | Dako Herceptest | HercepTest scoring criteria |
| Sanchez et al. | Retrospective cohort | Dako Herceptest | HercepTest scoring criteria |
| Koeppen et al. | Case control | Dako Herceptest | HercepTest scoring criteria |
| Osman et al. | Retrospective cohort | Dako Herceptest | HercepTest scoring criteria |
| Reese et al. | Case series | Zymed Laboratories, TAB 250 | Other |

Supplementary Table 2. Critical Appraisal Skills Programme checklist assessment

|  | Q1 | Q2 | Q3 | Q4 | Q5 | Q6 | Q7 | Q8 | Q9 | Q10 | Q11 |
| --- | --- | --- | --- | --- | --- | --- | --- | --- | --- | --- | --- |
| Balta et al. | Yes | Yes | No | No | Yes | Yes | Yes | No | Yes | Yes | Yes |
| Estephan et al. | Yes | Yes | Yes | Yes | Yes | Yes | Yes | Yes | Yes | Yes | Yes |
| Dag et al. | Yes | Yes | No | No | Yes | Yes | Yes | No | Yes | Yes | Yes |
| Peixoto et al. | Yes | Yes | Yes | No | Yes | Yes | Yes | Yes | Yes | Yes | Yes |
| Açikalin et al. | Yes | Yes | No | No | Yes | Yes | No | No | Yes | Yes | Yes |
| Zahir et al. | Yes | Yes | No | Yes | Yes | Yes | Yes | Yes | Yes | Yes | Yes |
| Tobiume et al. | Yes | Yes | No | Yes | Yes | Yes | Yes | Yes | Yes | Yes | Yes |
| Zhang et al. | Yes | Yes | No | No | Yes | Yes | Yes | Yes | Yes | Yes | Yes |
| Ramieri et al. | Yes | Yes | Yes | Yes | Yes | Yes | No | Yes | Yes | Yes | Yes |
| Montironi et al. | Yes | Yes | No | No | Yes | Yes | Yes | No | Yes | Yes | Yes |
| Nishio et al. | Yes | Yes | No | Yes | Yes | Yes | Yes | Yes | Yes | Yes | Yes |
| Lara et al. | Yes | Yes | Yes | No | Yes | Yes | Yes | Yes | Yes | Yes | Yes |
| Sanchez et al. | Yes | Yes | Yes | No | Yes | Yes | Yes | Yes | Yes | Yes | Yes |
| Koeppen et al. | Yes | Yes | No | Yes | Yes | Yes | Yes | Yes | Yes | Yes | Yes |
| Osman et al. | Yes | Yes | No | No | Yes | Yes | Yes | Yes | Yes | Yes | Yes |
| Reese et al. | Yes | Yes | No | No | Yes | Yes | Yes | Yes | Yes | Yes | Yes |

Supplementary material 1. Search algorithms

PubMed

(“Prostatic Neoplasms”[Mesh] OR “Prostate Cance*”[tiab] OR “Prostate Carcinom*”[tiab] OR “Prostate Adenocarcinom*”[tiab] OR “Prostatic Cance*”[tiab] OR “Prostatic Carcinom*”[tiab] OR “Prostatic Adenocarcinom*”[tiab]) AND (“Immunohistochemistry”[Mesh] OR “Immunohistochem*”[tiab] OR “IHC”[tiab]) AND (“Receptor, ErbB-2”[Mesh] OR “HER2”[tiab] OR “HER 2”[tiab])

WOS

(“Prostatic Neoplas*” OR “Prostate Neoplas*” OR “Prostate Cance*” OR “Prostate Carcinom*” OR “Prostate Adenocarcinom*” OR “Prostatic Cance*” OR “Prostatic Carcinom*” OR “Prostatic Adenocarcinom*”) AND (“Immunohistochem*” OR “IHC”) AND (“ErbB-2” or “ErbB2” or “ErbB 2” OR “HER2” or “HER 2”)

Medline

Prostatic Neoplasms/ and Immunohistochemistry/ and Receptor, ErbB-2/

Scopus

(“Prostatic Neoplas*” OR “Prostate Neoplas*” OR “Prostate Cance*” OR “Prostate Carcinom*” OR “Prostate Adenocarcinom*” OR “Prostatic Cance*” OR “Prostatic Carcinom*” OR “Prostatic Adenocarcinom*”) AND (“Immunohistochem*” OR “IHC”) AND (“ErbB-2” or “ErbB2” or “ErbB 2” OR “HER2” or “HER 2”)
